# Clinical Thermometry

**Published:** 1875-05-01

**Authors:** B. Kraus


### CLINICAL THERMOMETRY.

FROM THE "COMPENDIUM DER NEUEREN MEDICINISCHEN WISSENSCHAFTEN," BY DR. B. KRAUS.

*Condensed and translated by Dr. H. Gradle.*

### HISTORY AND SCOPE OF CLINICAL THERMOMETRY.

PROMINENT amongst the physical signs of disease, we find *animal heat*, the exact determination of which is of the greatest value in the diagnosis, the course, the prognosis, the therapeutic indications—in short, the rational treatment of disease. The importance of animal heat was known to the physicians of antiquity, and ever since their time has it been more or less appreciated. The first thermometric instrument was invented and constructed by Sanctorious, professor of medicine at Padua († 1638). After the lapse of more than a century, Boerhave made use again of the thermometer, after it had been neglected for clinical purposes for this period of time. In the year 1797 there appeared a work by James Currie, who based his therapeutic indications and success on thermometric observations. But to De Haen, the renowned professor of the clinic at Vienna, belongs the honor of being the founder of clinical thermometry. It was he who introduced the study of thermometry into pathology, who urged the employment of the thermometer at the bed-side, and adapted his treatment according to the state of the animal temperature. It was he who first considered the return of the animal warmth from the morbid to its normal standard as the true evidence of restored health. Andral has also recognized the importance of thermometry in pathology, and formulated in the year 1841 a series of definite laws on the temperature in disease.

Baerensprung, Traube, and especially Wunderlich, have within the last few years introduced thermometry into practical medicine, as they were the first who furnished careful, regular, and complete observations on the relation of temperature with diseases in their course, duration, and termination. Wunderlich especially deserves credit for his exact investigations, on the strength of which he has founded on clinical thermometry as reliable a method of diagnosis as on auscultation and percussion, and this method has attained such a reliability that, ahead of all others, it indicates the existence, degree, and importance of a disturbance of the system. By Henri Roger the thermometer has been employed in diseases of children, and a series of scientific observations on temperature in the different stages of childhood and its various diseases have been published (*Arch. Gen. de Med.*, 1844-5). Surgery, also, has resorted to the thermometer. Hunter first studied the temperature in surgical diseases; his example was followed by Thomson, Breschet, Bequerel, and finally by Demarquay, who has made experiments on the state of the temperature during pain, hæmorrhage, the ligation of arteries, traumatic inflammations, poisoning, etc. The same surgeon, as well as Dumeril, Lacointe, and many others, has examined the influence of therapeutic agents on the animal heat.

Clinical thermometry, says Wunderlich, is an objective physical method of examination, furnishing numerical evidence of a sufficiently sensitive nature to indicate progressively the changes in the organism.

Many physicians have placed thermometry, as a means of diagnosis, above percussion and auscultation; though this is hardly applicable in all cases. That much is certain, that it yields a diagnostic basis, possessing physical exactness, and independent of the estimation or the practice and acuteness of the senses of the observer.

The determination of temperature is all the more important since, as Wunderlich correctly affirms, it is at present the only exact expression, readily observable and with sufficient minuteness, of the total alteration of the organism; hence, by following it through the course of numerous cases of the same disease, the questions can be solved whether there are pathological processes obeying definite laws in their course, what deviations from this regular course occur, and by what they are caused.

The normal temperature of the human body, almost independent of the medium in which it exists, is in itself no sign of health, though the maintenance at its normal point under different influences, as hunger, cold, bodily and mental labor, motion, etc., if they do not reach such a degree as to cause a disturbance inevitably, can be considered as evidence of a sound constitution. It is true there are pathological conditions not resulting in a measurable alteration of the animal heat—apyretic diseases—but disease exists whenever the temperature exceeds the physiological limits, and *cæteris paribus* proportionately to the extent of the deviation. By one single thermometric examination we can merely decide whether the affection is or is not febrile; occasionally a diagnosis of the morbid variety can be formed from the observation of the first few days. More frequently we exclude with absolute certainty, by means of the temperature, diseases simulated by the other symptoms. In other cases of a doubtful nature, voluntary simulation can be thus detected. But also the different individual modifications of the affection, the transition from one stage to another, the time of exacerbation or remission, the development of complications, the severity of the morbid process can be recognized earlier and more precisely by the thermometric method than by any other. As soon as a variation from the ordinary thermometric course of any disease is observed, we have received an important warning, and oftentimes the first, bidding us to search for the cause of this irregularity, thus leading not rarely to the discovery of unknown complications. At the time of convalescence, also, the temperature is the best index of recovery. Finally, the animal warmth affords us the best guide as to the efficacy or want of effect of our therapeutic procedures.

In concluding this chapter, there is yet to mention that the fear that thermometric observations could not be resorted to in private practice on account of the opposition of the patients, is not warranted after the experience in this direction with the greater part of the public. Similar apprehensions were entertained when auscultation and percussion came into use, but now every practitioner observes that patients are often not satisfied unless thoroughly examined, even more than is necessary, at each visit; and similarly thermometry, after the public has once understood its importance, will become to them a matter of course. No one can shun the slight expense of purchasing an instrument, and, as its application does not inconvenience the patient, opposition to its use can hardly be expected; even the remonstrances of children can easily be overcome with a little tact. At the same time the manner of application is so simple that any patient or nurse can manipulate it with ease, when it remains for the physician but to read off the number, which can certainly not consume much time even in large hospitals—the less as it enables the physician to dispense with other modes of examination and avoid unnecessary questioning.

PROPERTIES OF A THERMOMETER FOR MEDICAL PURPOSES.

The estimation of temperature by means of the hand is a very unreliable method, as the hand, on account of its shape, cannot adapt itself to all parts, besides it adds or abstracts warmth from the place of contact. The hand appreciates only the amount of heat it receives or loses in a given time; in other words, the difference between its temperature and that of the object examined. Thus cold can be received if the body is cooler than the hand, even though it be of a temperature above the normal point. The use of the hand gives us, therefore, but an imperfect idea of the warmth of a body, and a hint whether a more accurate determination is indicated.

For the latter purpose we require a reliable, sensitive, easily transportable and solid instrument, properties mostly possessed by the mercury thermometer. Such a thermometer, according to Wunderlich, is to be of the following description: The mercury reservoir is to have a diameter of one-third to three-fourths centimetre; if larger it loses its sensitiveness; if smaller it cannot adapt itself well to the surface of the parts. The spherical shape is preferable to the cylinder for use in the axilla; at least, the reservoir, if cylindrical, is not to possess too great a length. For the rectum and vagina a conical reservoir tapering downwards appears most suitable. A hemisphere with flat bottom has been recommended for observation of external surfaces, but gives poor results. The glass of the reservoir should neither be too thin, on account of its fragility, nor too thick, as this would reduce the sensitiveness. The tube of the instrument is to possess a uniform calibre, so small as to enable the unaided eye to divide the space between two-tenth degrees into halves and quarters. The tube is to be of such a length that the degrees ordinarily observed are at a distance of at least twelve centimetres from the bulb to permit an easy observation of the instrument while in place.* For facil-

* This description does not refer to self-registering instruments.

ity of handling, however, the entire length ought to be limited, not exceeding much the expansion of the mercury corresponding to the degrees usually attained on man. It suffices, if the scale covers but the degrees between 32.5–45° C. (90–113° F.), perhaps including also those down to 75° F. for bathing purposes, the highest being at a distance of about twelve centimetres from the bulb. A division into one-fifth degrees is sufficient for ordinary purposes.

The accuracy of the thermometer depends more on the uniform construction of the scale than on the correctness of its numbers, which latter can be regulated by comparison with a standard. If the difference between it and the standard is the same at all heights, the mere addition or subtraction of the same corrects the result perfectly. Occasional testing is to be recommended for the best thermometers for medical purposes, for the glass suffers light changes of its molecular architecture, which can affect the calibre, especially of the bulb, so that after a time the difference may amount to several tenths degrees; perhaps, also, the compression in handling the bulb, etc., may be a factor of the result. Whenever the temperature observed seems unaccounted for, a speedy testing of the thermometer is advisable.

An exceptional accuracy is attained by the use of the so-called *metastatic thermometers of Walferdin*, while for some investigations the thermo-electric apparatus appears most serviceable. The latter is constructed on the principle that an electric current is induced in a metal ring consisting of two different metals, whenever the joints are subjected to different temperatures, even though this difference be very considerable, and that this current can be indicated by the magnetic needle. Bequerel has used such an apparatus for physiological purposes. Wunderlich considers it superfluous for clinical observations. The thermograph of Marey has not yet been tested at the bedside, and will hardly ever be; it is intended to give a graphic trace of the thermometric course.

### PLACE OF APPLICATION OF THE THERMOMETER.

While the thermometer can be applied to any part of the body, the most suitable place varies with the purpose of the investigation. If it be the intention to measure the temperature of a limited part, the instrument is of course to be applied at that place. If this is superficial, a thermo-electric apparatus is preferable, as the mercury thermometer is cooled off by the surrounding air unless protected. The instrument must be so applied as to be surrounded by the parts. In most cases the closed axilla appears the most suitable, as its examination does not prove inconvenient to the patient. But in emaciated or restless patients the result is not accurate; besides the temperature is always lower, and slight oscillations are not as easily observed in the axilla as in the mucous cavities, mouth, rectum, vagina, or uterus. Holding the thermometer in the closed hand is a method of no value in the determination of the bodily temperature, unless we wish to compare the extremities with each other or with the trunk.

Insertion into the buccal cavity is not a very accurate method, as the instrument is somewhat cooled off by the inspired air; besides it interferes with the respiration of some patients, while in others it produces a tendency to vomiting. Other places of application are therefore preferable.

A more accurate plan than either of the preceding is to introduce the thermometer into the rectum, against which, however, patients frequently protest. The method is objectionable on æsthetic grounds. By the irritation defecation is occasionally induced, and an erroneous result is also obtained if the instrument enters fæcal masses. It is said, besides, that the temperature varies according to the depth, a correct result being obtained only if the bulb passes in as far as the prostate (Hunter).

Contractions of the rectum are sometimes observed altering the temperature (Billroth). It is, however, advisable to resort to the rectum as a place of application in diseases and stages in which great accuracy is necessary, and to return to the axilla when such precision becomes less important, of course noting the place of application in the tables.

The insertion into the vagina is a more accurate method than the previous, but not often applicable. It is, of course, requisite whenever the temperature of the internal genital organs is to be determined.

Winkl, Schroeder, Traube, and W. Schlesinger, of Vienna, have also made thermometric observations in the cavity of the uterus, partly to study the temperature of the female system during menstruation, pregnancy, parturition, etc., partly to determine the influence of pathological calorification on febrile temperature, and finally to diagnosticate pregnancy in the earlier months before other symptoms are developed. The possibility of such a diagnosis is warranted by the fact that the child has a temperature of its own slightly higher than that of the mother, and that the uterus is found warmer than the vagina in pregnancy, probably from the higher temperature of the fœtus. The main difficulty in these investigations is the construction of a suitable instrument, as the ordinary thermometers cannot be introduced into the womb.* Schlesinger demonstrated in the "Gesellschaft der Aertze in Wien" (session of March 6th, 1874), a thermometer for this purpose, curved at one end, the other having attached to it a scale divided into tenths of degrees. On account of its fragility, the mercury reservoir is surrounded by a metal covering, permitting its introduction into the uterus without danger. In order to avoid average temperatures, the bulb was reduced in length from three and one-half to about two centimetres, so that the cervical and corporeal cavities can be measured separately.

By means of this instrument, Schlesinger has found the difference between axilla and vagina to amount to 0.21° C. (0.38° F.), between vagina and uterus to 0.16° C. (0.29° F.), in the non-pregnant state. These results agree with those of Schroeder and Winkl, who also observed a higher temperature in the uterus than in the vagina, both in the pregnant and non-pregnant state. Further on, he found both uterus and vagina warmer than the rectum, the latter by about 0.05° C. (0.09° F.). Between the cavity of the uterus and the cervix there is also a slight difference in favor of the former.

* Not so with self-registering instruments.

